# Critical items for assessing risk of lung and colorectal cancer in primary care: a Delphi study

**DOI:** 10.3399/bjgp14X681001

**Published:** 2014-07-25

**Authors:** Gemma Mansell, Mark Shapley, Danielle van der Windt, Tom Sanders, Paul Little

**Affiliations:** Arthritis Research UK Primary Care Centre, Research Institute for Primary Care & Health Sciences, Keele University, Keele.; Arthritis Research UK Primary Care Centre, Research Institute for Primary Care & Health Sciences, Keele University, Keele.; Arthritis Research UK Primary Care Centre, Research Institute for Primary Care & Health Sciences, Keele University, Keele.; Arthritis Research UK Primary Care Centre, Research Institute for Primary Care & Health Sciences, Keele University, Keele.; Primary Medical Care, Aldermoor Health Centre, Southampton.

**Keywords:** Delphi technique, neoplasms, primary health care, referral and consultation

## Abstract

**Background:**

Patients with lung or colorectal cancer often present late and have a poor prognosis. Identifying diagnostic indicators to optimally assess the risk of these cancers in primary care would support early identification and timely referral for patients at increased risk.

**Aim:**

To obtain consensus regarding potential diagnostic indicators that are important for assessing the risk of lung or colorectal cancer in primary care consulters presenting with lung or abdominal symptoms.

**Design and setting:**

A Delphi study was conducted with 28 participants from primary and secondary care and academic settings in the UK and Europe.

**Method:**

Indicators were obtained from systematic reviews, recent primary studies and consultation with experts prior to the Delphi study being conducted. Over three rounds, participants rated each diagnostic indicator in terms of its importance, ranked them in order of importance, and rated each item as crucial or not crucial to assess during a GP consultation.

**Results:**

The final round resulted in 25 items remaining for each type of cancer, including established cancer symptoms such as rectal bleeding for colorectal cancer and haemoptysis for lung cancer, but also less frequently used indicators such as patients’ concerns about cancer.

**Conclusion:**

This study highlights the items clinicians feel would be most crucial to include in the clinical assessment of primary care patients, a number of which have rarely been noted in the previous literature. Their importance in assessing the risk of lung or colorectal cancer will be tested as part of a large prospective cohort study (CANDID).

## INTRODUCTION

Lung and colorectal cancer are two of the most common cancers seen in UK primary care. Colorectal cancer is the second most commonly diagnosed cancer in the UK, with almost 40 000 new cases per year documented in 2009.^1^ Lung cancer deaths in England and Wales represent 22% of the total mortality from cancer.^2^ Patients in the UK have been reported to present later and do worse than in other countries,^3^ raising the issues of early identification and referral in primary care, prompt diagnosis, and effective treatment. However, most symptoms that may indicate cancer are common in primary care and are associated with a very low cancer risk. Referrals based on these symptoms can lead to unnecessary anxiety and investigations for patients at low risk, and unnecessary use of secondary care services.

One way of expressing a patient’s risk of cancer is to use a positive predictive value (PPV), which is the proportion of people with a particular diagnostic indicator who go on to develop cancer.^4^ There is a lack of evidence for the predictive values of lung or colorectal cancer symptoms, signs, and test results derived from high-quality, prospective primary care cohorts.^5^–^7^ In consulting populations the proportion with cancer is lower in primary care than in secondary care, and consequently the PPVs for this population are lower. Recent systematic reviews have identified few symptoms, signs, and test results in primary care that have a PPV of above 5%,^4^,^8^ meaning that many have a relatively low risk of being cancer-related.

Clinical prediction rules (CPRs), derived from prospective data collection and consisting of the combination of symptoms, signs, and test results most strongly associated with cancer risk, may be the most robust and reliable way to inform decisions regarding further investigations and onward referral. Existing CPRs for assessing cancer risk have often been developed in secondary care populations,^9^ meaning they may be less accurate in primary care, and others^10^,^11^ have been derived using only routinely collected data, which may not provide valid, standardised information in the way that a study designed specifically to collect information on diagnostic indicators would do. A large, prospective primary care-based cohort study (CANcer DIagnosis Decision rules, CANDID) has therefore been designed to derive prediction rules to support the early diagnosis of lung cancer and colorectal cancer in primary care. This Delphi study represents the first phase of this project, and aims to obtain consensus regarding potential diagnostic indicators that are important for assessing risk of lung and colorectal cancer in primary care consulters presenting with symptoms of possible oncological significance, with a particular emphasis on factors not already supported by evidence from previous research.

**How this fits in**

It is known that patients with lung or colorectal cancer often present late and have a poor prognosis. Developing clinical prediction rules for primary care may help ensure prompt and appropriate referral. This Delphi study has generated a list of diagnostic indicators that primary care clinicians feel are crucial to assess in a consultation with a patient presenting with symptoms that may be indicative of lung or colorectal cancer. These items will be tested as part of a large, prospective primary care-based cohort study, which will help identify the most important factors for clinicians to focus on when presented with a patient with lung or colorectal symptoms.

## METHOD

A modified Delphi study^12^ was designed to achieve consensus regarding key diagnostic indicators in the identification of primary care patients at risk of lung or colorectal cancer. The Delphi is described as modified because participants were presented with a list of items to consider, rather than generating their own items,^12^ although there was an option to do this within the study. The Delphi process was anonymised, with panel members unaware of the identity of other members, and responses kept anonymous.

### Identification of Delphi panel members

A panel of national and international cancer experts, researchers, and clinicians from primary and secondary care were invited to participate. Participants were identified through the West Midlands North primary care research network, as authors of relevant research publications identified from a scoping search, or via personal networks of the CANDID study team. Seventy-three potential participants were initially identified, with the aim to recruit approximately 20 panel members. There are no set guidelines for deciding on the optimum number of Delphi participants as this is likely to change depending on the purpose of the Delphi.^13^

### Design of the Delphi study

All study materials were sent to participants via email. Participants were informed that their responses would be anonymised. The Delphi rounds were constructed using web-based survey software (Survey Monkey), which participants accessed via a weblink included in the email. The study was conducted between August and November 2012.

**Figure 1.**
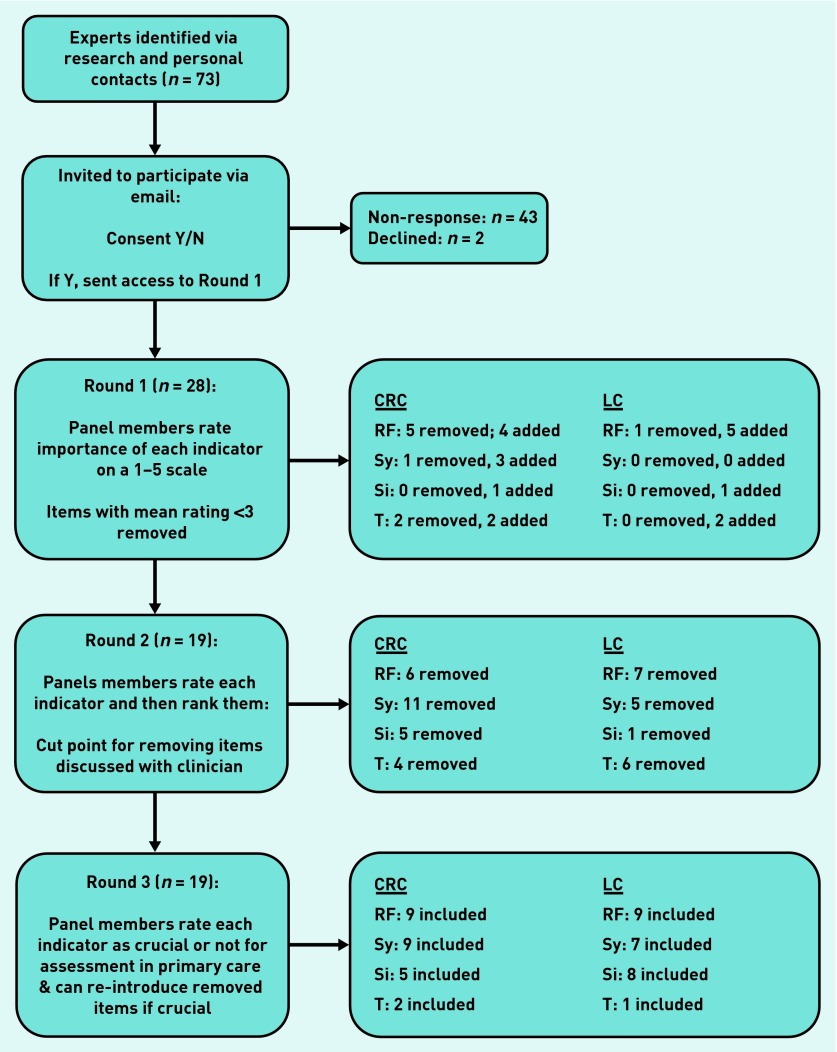
***Flowchart of how information was collected over the three Delphi rounds. CRC = colorectal cancer. LC = lung cancer. RF = risk factor. Sy = symptom. Si = sign. T = test.***

An overview of data collection throughout the study can be found in Figure 1. The first round of the Delphi contained the consent form, and participants could only access the survey if they consented to take part. Participants could also choose whether they wanted to answer questions relating to both types of cancer or only lung or colorectal cancer. Items to be included in the first round of the Delphi were identified from a scoping search of the literature to locate previously conducted systematic reviews and key primary studies that had identified factors predictive of lung or colorectal cancer in primary care (for example). 4,8,14,15 Other items were identified by members of the study team and through discussion with expert clinicians.

Round 1 involved participants rating the identified diagnostic indicators on a 5-point Likert scale, ranging from ‘very unimportant’ to ‘very important’. The indicators were split into ‘key risk factors’, ‘symptoms’, ‘signs’ and ‘tests’ for lung and colorectal cancer. Participants could also add up to three additional indicators that they felt were missing from the current lists, and were asked to give a reason why they thought that item was important. Participants were also asked to provide information on their occupation (clinical or non-clinical) and the number of years of experience they had, to allow a description of the key characteristics of the panel. Any indicators which received a mean score of <3.0 were removed in the second round, but all new indicators were included. The cut-off point of 3.0 was set to include most items deemed important at this early stage.

Round 2 involved participants ranking each of the indicators in terms of how important they felt they were, with a score of 1 given to the most important indicator and the highest number to the least important indicator in each list. Summated scores generated a ranking within each list of key risk factors, symptoms, signs, and tests. The number of indicators to take forward to the final round was decided by the study team through discussions with a GP, who provided independent clinical input when deciding on the optimal cutoff for removing indicators from each section. To allow final decisions regarding items that should be maintained in the clinical assessment, participants in the final round were asked which of the remaining indicators were crucial or not crucial to assess in a 10-minute GP consultation with patients presenting with either lung or abdominal symptoms suggestive of cancer. Participants could reintroduce a limited number of crucial items that had been removed over the previous rounds, providing a reason for reintroduction of the item.

The rounds were sent out to participants every 4 weeks, with a reminder email being sent out 2 weeks after the initial email. All participants were sent an anonymised summary feedback report at the end of every round to inform them of study progress.

## RESULTS

### Characteristics of the Delphi panel

Of the 73 people originally invited to participate, 28 responded to round 1, 19 to round 2 and 19 to round 3. The participants described themselves mostly as GPs working in a clinical capacity (*n* = 25); two described themselves as non-clinical, and one as a specialist. Across the panel there was a mean of 22.7 years of experience (range 5–42 years).

### Results of round 1

The results of each round are presented in Table 1 and Table 2 for colorectal cancer and lung cancer, respectively, which also show which items were removed and added. In round 1, few items were removed for either cancer as almost all items received a mean score of ≥3.0, and a number of items were added. Some of these added items were specific to the type of cancer, such as melaena as a sign for colorectal cancer and Horner’s syndrome as a sign for lung cancer. Others were more generic, including jaundice and patients’ concerns regarding their symptoms for either type of cancer.

**Table 1.** Inclusion and exclusion of diagnostic indicators at each Delphi round for colorectal cancer

**Diagnostic indicator**	**Round 1 (mean score for importance)**	**Round 2 (mean rank score)**	**Round 3 (crucial)**
**Key risk factor**			
Familial polyposis coli^a^	4.92	2.44	Y
First-degree relative with colorectal cancer <50 years^a^	4.85	3.61	Y
Age^a^	4.54	3.17	Y
More than 10 polyps^a^	4.38	6.67	Y
First-degree relative with polyps <50 years^a^	4.27	5.94	Y
Second attendance with the same symptom^a^	4.23	9.28	Y
Inflammatory bowel disease^a^	4.15	6.72	Y
Benign polyps	3.73	10.28	N^b^
Smoking status	3.35	10.83	N^b^
First-degree relative with other type of cancer	3.42	11.89^b^	
History of endometrial cancer	3.23	12.56^b^	
Last consultation with a GP >6 months ago	3.15	13.56^b^	
Alcohol intake	3.00	13.78^b^	
Socioeconomic status^b^	2.92^b^		
Ethnicity^b^	2.88^b^		
Diabetes mellitus^b^	2.73^b^		
Sex^b^	2.65^b^		
Occupational history^b^	2.46^b^		
Progression of symptoms^a^,^c^		5.33	Y
Patient thinks they have colorectal cancer^a^,^c^		10.94	Y
Last consultation with a GP >5 years ago^c^		11.00	
Patient attends with an adult family member^c^		15.00	

**Symptom**			
Rectal bleeding: blood mixed with stool^a^	4.73	1.88	Y
Bowel symptoms: change in bowel habit^a^	4.73	3.24	Y
Unintentional loss of weight reported by patient^a^	4.73	4.82	Y
Rectal bleeding: type^a^,^d^	4.50	4.06	Y
Symptom duration^a^,^e^	4.46	8.35	Y
Bowel symptoms: tenesmus^a^	4.08	8.76	Y
Bowel symptoms: urgency	3.88	9.71	N^b^
Bowel symptoms: incomplete emptying^a^	3.88	9.29	Y
Bowel symptoms: diarrhoea^a^	3.81	8.24	Y
Fatigue	3.77	15.35^b^	
Loss of appetite	3.73	13.35^b^	
Abdominal pain	3.69	11.24^b^	
Bowel symptoms: nocturnal symptoms	3.62	11.47^b^	
Bowel symptoms: constipation	3.42	14.94^b^	
Distension	3.42	14.88^b^	
Discomfort	3.27	16.65^b^	
Peri-anal symptoms	3.23	14.47^b^	
Bloating	3.12	16.53^b^	
Nausea^b^	2.88^b^		
Jaundice^a^,^c^		8.24	Y
Mucus in stool^c^		17.12^b^	
Wet wind^c^		18.41^b^	

**Sign**			
Rectal mass^a^	4.96	1.88	Y
Abdominal mass^a^	4.85	3.12	Y
Cachexia^a^	4.81	4.53	Y
Objective loss of weight^a^	4.65	4.59	Y
Rectal examination: blood on glove^a^	4.27	5.29	Y
Ascites	4.54	6.00^b^	
Hepatomegaly	4.31	6.12^b^	
Pale conjunctivae	3.73	8.29^b^	
Lymphadenopathy	3.73	7.76^b^	
Melaena^c^	7.41^b^		

**Test**			
Iron deficiency anaemia^a^	4.77	1.12	Y
Anaemia	4.15	2.94	N^b^
Positive faecal occult blood test^a^	3.96	3.53	Y
Raised erythrocyte sedimentation rate	3.73	4.18^b^	
Screened for colorectal cancer in the last 2 years	3.54	4.76^b^	
High white cell count^b^	2.54^b^		
Raised glucose level^b^	2.35^b^		
Disturbed liver function tests^c^		5.06^b^	
Hypercalcaemia^c^		6.41^b^	

a Included in round 3.

b Excluded in round 2.

c Added by participants in round 1.

d Refers to characteristics of the rectal bleeding experienced (for example, dark versus bright red blood, whether the blood was noticed in the toilet pan or on the toilet paper.

e Refers to whether the symptom was present for a long period of time as opposed to only a short period.

**Table 2.** Inclusion and exclusion of diagnostic indicators at each Delphi round for lung cancer

**Diagnostic indicator**	**Round 1 (mean score for importance)**	**Round 2 (mean rank score)**	**Round 3 (crucial)**
**Key risk factor**			
Smoking status^a^	5.00	1.13	Y
Occupational exposure^a^	4.54	4.50	Y
Age^a^	4.33	3.31	Y
Second attendance with the same symptom^a^	4.25	8.25	Y
History of chronic obstructive pulmonary disease^a^	4.13	6.81	Y
Second-hand smoke exposure^a^	4.04	7.00	Y
Socioeconomic status	3.71	7.19	N^b^
Family history (first-degree relative)^a^	3.58	9.56	Y
Last consultation with a GP >6 months ago	3.46	12.88^b^	
History of idiopathic pulmonary fibrosis	3.42	12.75^b^	
History of tuberculosis	3.33	13.81^b^	
Sex	3.25	11.19^b^	
Alcohol intake	3.17	11.75^b^	
Ethnicity^b^	2.79^b^		
Last consultation with a GP >5 years ago^c^		10.38	N^b^
Patient thinks they may have lung cancer^a^,^c^		11.13	Y
Patient attends with an adult family member^c^		15.75^b^	
Localisation of pain^c^		12.63^b^	
Reduced exercise tolerance^a^,^c^			Y

**Symptom**			
Haemoptysisa	4.96	1.25	Y
Unintentional loss of weight reported by patient^a^	4.79	4.44	Y
Symptom duration^a^,^d^	4.25	6.19	Y
Cough^a^	4.17	3.00	Y
Hoarseness^a^	4.13	6.06	Y
Dyspnoea^a^	4.00	4.38	Y
Chest pain^a^	3.79	6.69	Y
Bone pain	3.92	9.88^b^	
Loss of appetite	3.88	7.88^b^	
Back pain	3.88	10.56^b^	
Fatigue	3.88	8.81^b^	
Shoulder pain	3.75	8.88^b^	

**Sign**			
Cachexia^a^	4.71	3.94	Y
Pleural mass^a^	4.67		
Pleural effusion^a^	4.67	3.69	Y
Superior vena cava obstruction^a^	4.58	3.31	Y
Objective weight loss^a^	4.54	4.5	Y
Lymphadenopathy^a^	4.25	5.94	Y
Stridor^a^	4.21	6.00	Y
Finger clubbing	4.00	7.31^b^	
Horner's syndrome^a^,^c^		6.50	Y

**Test**			
Abnormal chest X-ray^a^	4.79	1.06	Y
Abnormal sputum cytology	4.13	3.75	N^b^
Anaemia	3.71	3.69	N^b^
Raised erythrocyte sedimentation rate	3.75	4.63^b^	
Raised C-reactive protein	3.5	5.56^b^	
Abnormal spirometry	3.33	6.06^b^	
Thrombocytosis	3.25	7.31^b^	
Hypercalcaemia^c^		6.81^b^	
Disturbed liver function tests^c^		6.13^b^	

a Included in round 3.

b Excluded in round 2.

c Added by participants in round 1.

d Refers to whether the symptom was present for a long period of time as opposed to only a short period.

### Results of round 2

Based on the summated rank scores described in the Method section, abnormal chest X-ray, smoking status, and haemoptysis were ranked most highly for lung cancer and iron deficiency anaemia, blood mixed with stool and rectal mass ranked most highly for colorectal cancer. Based on the importance given to each item, the number of items in each section (risk factors, symptoms, signs, and tests) was reduced by approximately 50%, resulting in a total number of 29 potential diagnostic indicators for colorectal cancer and 28 for lung cancer (Tables 1 and 2). The rank scores given to the items were very similar across the items for the second round, meaning that it was difficult to judge where to place a cut-off based on the rank scores alone.

### Results of round 3

Similar to round 1, few items were removed for either type of cancer in this final round, with nearly all indicators being regarded as crucial. Participants could reintroduce previously removed items, but as none of these were suggested for reinclusion more than once, no items were re-entered. The final items for lung cancer included nine risk factors, seven symptoms, eight signs, and one test, while the final list of items for colorectal cancer was made up of nine risk factors, nine symptoms, five signs, and two tests (Figure 1).

## DISCUSSION

### Summary

This Delphi study generated consensus among cancer experts and primary care clinicians regarding the diagnostic indicators that are most important to assess during a GP consultation to more reliably estimate a patient’s risk of lung or colorectal cancer. The study identified a list of 25 potential indicators each for lung cancer and colorectal cancer, which will provide a guide to the design of web-based data collection forms to be completed by GPs participating in the main CANDID study.

### Strengths and limitations

As this study included a mostly primary care-based panel, the authors are confident that the results reflect what GPs perceive as important in everyday clinical practice. This is a strength as the CPRs will be designed and tested in a primary care-based study. Limited data collection on the characteristics of participants means that a full assessment concerning generalisability cannot be made. The consensus represented by the varied clinical experience and academic knowledge of the panel is supported by the literature and also includes novel items that may potentially lead to an improvement in the early identification of cancer.

Although the items were initially chosen from the published literature, further suggestions were made by the study team members and Delphi panel members, and the choice of cut-offs for each round was, although *a priori* defined, also based on subjective clinical judgement. The use of an independent GP to provide input regarding the cut points after round 2 had the advantage of weighing towards use in practice, although this may have introduced subjectivity. The final round therefore allowed reintroduction of crucial items and consensus regarding the final outcome.

### Comparison with existing literature

Consensus was reached on the classic alarm symptoms by which lung and colorectal cancer present, such as haemoptysis and rectal bleeding, although the number of acknowledged symptoms highlights the vagueness and variety of clinical presentations. Some of the crucial symptoms that were eventually decided on were subtypes of classic symptoms (for example, change in bowel habit) into more specific types (for example, diarrhoea as opposed to constipation), and may reflect clinical experience concerning the incidence of the more generic symptoms in primary care populations and their low predictive value for cancer. These findings are consistent with results from other primary care-based studies.^8^,^10^ Patients who present with classic alarm symptoms may have a lower risk of mortality than those who do not,^16^ possibly because these symptoms are more easily recognisable as potential cancer symptoms. There may be some inconsistency with patients’ ideas concerning key symptoms, with one study of symptoms for colorectal cancer^17^ finding rectal bleeding, change in bowel habit, and weight loss being commonly reported symptoms, along with pain, fatigue, and ‘general indisposition’ or feeling of being unwell. Pain and fatigue have been reported as warning signs for cancer,^18^ but with very low PPVs.

Well-known cancer-specific risk factors were classified by the panel as crucial (for example, familial polyposis coli for colorectal cancer and chronic obstructive pulmonary disease for lung cancer). With more generic risk factors, some appeared to be judged as crucial for both cancers (for example, age), others were seen as crucial for one cancer but not the other (for example, smoking for lung cancer only) and others were not judged to be crucial for either type of cancer (for example, socioeconomic status). This is likely to be reflective of the make-up of the panel who were mostly GPs with extended clinical experience. A qualitative study of GPs^19^ found this experience to be a key factor in judging the possibility of cancer in a patient, and also highlighted the importance of interpersonal awareness and how subtle changes in the way a patient talks or behaves may be indicative of a more serious condition.

Symptom duration was present in the first round of the Delphi as an aid to judging cancer probability, although this may represent a high probability of cancer if the duration is short (6 weeks for example) or a low probability if it is long (years). It has been noted that time as a diagnostic tool should be used when the benefits of the delay outweigh any potential harm to the patient,^20^ and that it is useful when confronted with vague, common symptoms which characterise many presentations of possible cancer. The importance of ‘second attendance with the same symptom’ for lung and colorectal cancer and the introduction by participants of ‘progression of symptoms’ for colorectal cancer represents further use of time as a diagnostic tool within primary care. The absence of this item for lung cancer may reflect the more progressive course of this illness. An additional crucial factor introduced by participants was that of patients’ concerns regarding risk of cancer, representing a patient-centred approach in primary care. Fear of cancer has been found to be prevalent in presenting patients^21^ and GPs, through a fear of missing a diagnosis.^20^ These items have not been previously investigated in diagnostic studies, possibly because they are difficult to measure. The inclusion of these more subjective indicators could again be a result of the make-up of the panel.

### Implications for research and practice

The findings of this Delphi consensus study will inform a large primary care-based prospective cohort study (CANDID) by highlighting the items that clinicians feel are most important to include in the clinical assessment of primary care consulters. This study has also helped to highlight a number of potential indicators that have rarely been noted in the literature. Their importance in assessing the risk of lung and colorectal cancers will be tested as part of the CANDID study.
